# Robustness of apparent diffusion coefficient–based lymph node classification for diagnosis of prostate cancer metastasis

**DOI:** 10.1007/s00330-023-10406-8

**Published:** 2023-12-15

**Authors:** Benjamin Noto, Maria Eveslage, Katharina Auf der Springe, Anne Exler, Andreas Faldum, Walter Heindel, Stanislaw Milachowski, Wolfgang Roll, Michael Schäfers, Lars Stegger, Jochen Bauer

**Affiliations:** 1 Clinic for Radiology, University of Münster and University Hospital Münster, Münster, Germany; 2 Department of Nuclear Medicine, University Hospital Münster, Münster, Germany; 3 Institute of Biostatistics and Clinical Research, University of Münster, Münster, Germany; 4 West German Cancer Centre (WTZ), University Hospital Münster, Münster, Germany

**Keywords:** Prostatic neoplasms, Lymphatic metastasis, Diffusion magnetic resonance imaging, Magnetic resonance imaging, Positron emission tomography

## Abstract

**Objectives:**

The aim of this proof-of-principle study combining data analysis and computer simulation was to evaluate the robustness of apparent diffusion coefficient (ADC) values for lymph node classification in prostate cancer under conditions comparable to clinical practice.

**Materials and methods:**

To assess differences in ADC and inter-rater variability, ADC values of 359 lymph nodes in 101 patients undergoing simultaneous prostate-specific membrane antigen (PSMA)-PET/MRI were retrospectively measured by two blinded readers and compared in a node-by-node analysis with respect to lymph node status. In addition, a phantom and 13 patients with 86 lymph nodes were prospectively measured on two different MRI scanners to analyze inter-scanner agreement. To estimate the diagnostic quality of the ADC in real-world application, a computer simulation was used to emulate the blurring caused by scanner and reader variability. To account for intra-individual correlation, the statistical analyses and simulations were based on linear mixed models.

**Results:**

The mean ADC of lymph nodes showing PSMA signals in PET was markedly lower (0.77 × 10^−3^ mm^2^/s) compared to inconspicuous nodes (1.46 × 10^−3^ mm^2^/s, *p *< 0.001). High inter-reader agreement was observed for ADC measurements (ICC 0.93, 95%CI [0.92, 0.95]). Good inter-scanner agreement was observed in the phantom study and confirmed in vivo (ICC 0.89, 95%CI [0.84, 0.93]). With a median AUC of 0.95 (95%CI [0.92, 0.97]), the simulation study confirmed the diagnostic potential of ADC for lymph node classification in prostate cancer.

**Conclusion:**

Our model-based simulation approach implicates a high potential of ADC for lymph node classification in prostate cancer, even when inter-rater and inter-scanner variability are considered.

**Clinical relevance statement:**

The ADC value shows a high diagnostic potential for lymph node classification in prostate cancer. The robustness to scanner and reader variability implicates that this easy to measure and widely available method could be readily integrated into clinical routine.

**Key Points:**

*• The diagnostic value of the apparent diffusion coefficient (ADC) for lymph node classification in prostate cancer is unclear in the light of inter-rater and inter-scanner variability.*

*• Metastatic and inconspicuous lymph nodes differ significantly in ADC, resulting in a high diagnostic potential that is robust to inter-scanner and inter-rater variability.*

*• ADC has a high potential for lymph node classification in prostate cancer that is maintained under conditions comparable to clinical practice.*

**Graphical abstract:**

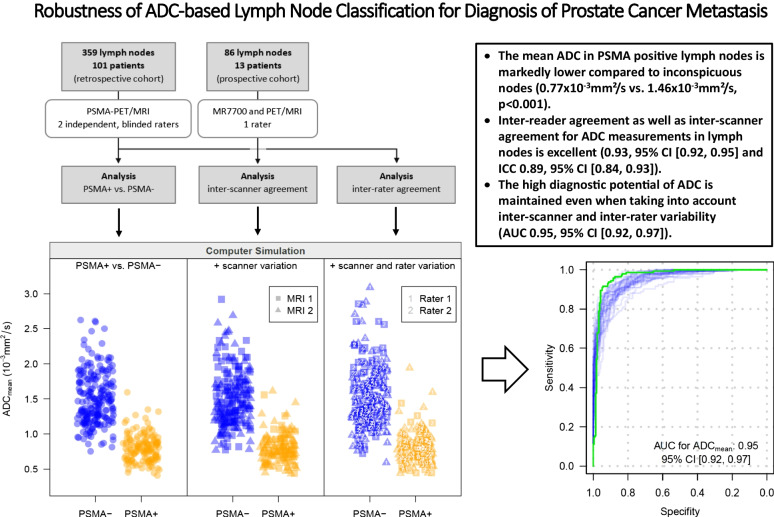

**Supplementary Information:**

The online version contains supplementary material available at 10.1007/s00330-023-10406-8.

## Introduction

Prostate cancer is the second most common cancer in men [1]. After local therapy, biochemical recurrence develops in about one-third of patients. The value of imaging in the setting of biochemical recurrence has been limited [2], mainly due to the poor performance of conventional imaging to detect lymph node metastases, which often are the only site of recurrence [3]. Compared to conventional cross-sectional imaging, PET targeting the prostate-specific membrane antigen (PSMA) demonstrates superior sensitivity for lymph node metastases [4–6]. The clinical value of improved imaging-based lymph node classification was demonstrated in a recent study showing increased event-free survival when PSMA-PET information on nodal metastases was accounted for in salvage radiation field planning [7].

Although PSMA-PET demonstrates high diagnostic accuracy in lymph node metastasis detection [4–6], it might not always be available, involves radiation exposure, and is associated with considerable costs [8]. Therefore, improving lymph node classification of more widely available imaging technologies is desirable.

Overcoming classical measurement of the short axis for lymph node classification, an MRI method that has shown promise is diffusion-weighted MRI (DWI). DWI depends on the differences in the movement of water and other small molecules based on Brownian motion, which can be quantified by apparent diffusion coefficient (ADC) maps. ADC values have been shown to be lower in many malignant lesions compared to benign tissue [9, 10]. Previous studies investigating the capacity of ADC for lymph node classification in prostate cancer produced conflicting results [11–17]. Also, the impact of inter-rater and inter-scanner variability on diagnostic accuracy of ADC has not been considered in those studies. Even if ADC values differed significantly between benign and malignant lymph nodes, a high inter-rater or inter-scanner variability in relation to this difference would reduce the diagnostic value and hinder transferability. Previous studies that report differences in ADC values depending on the status of lymph nodes therefore do not allow any direct conclusions to be drawn about the diagnostic quality of the ADC value under real-world conditions.

In this proof-of-concept study, we investigate the robustness of ADC-based lymph node classification in prostate cancer by analyses of the difference in ADC between malignant and benign lymph nodes on a node-by-node level. In a computer simulation approach, we combine these findings with results on inter-rater and inter-scanner variability to assess the diagnostic quality of ADC measurement for detection of lymph node metastasis under conditions comparable to clinical practice.

## Materials and methods

### Study design and patients

The presented study was approved by the local ethics committee (2018-643-f-S, 2021-766-f-S) and performed in accordance with the 1964 Declaration of Helsinki and its amendments. All measurements were performed between November 2016 and March 2022 at the University Hospital Münster, Germany.

**Fig. 1:**
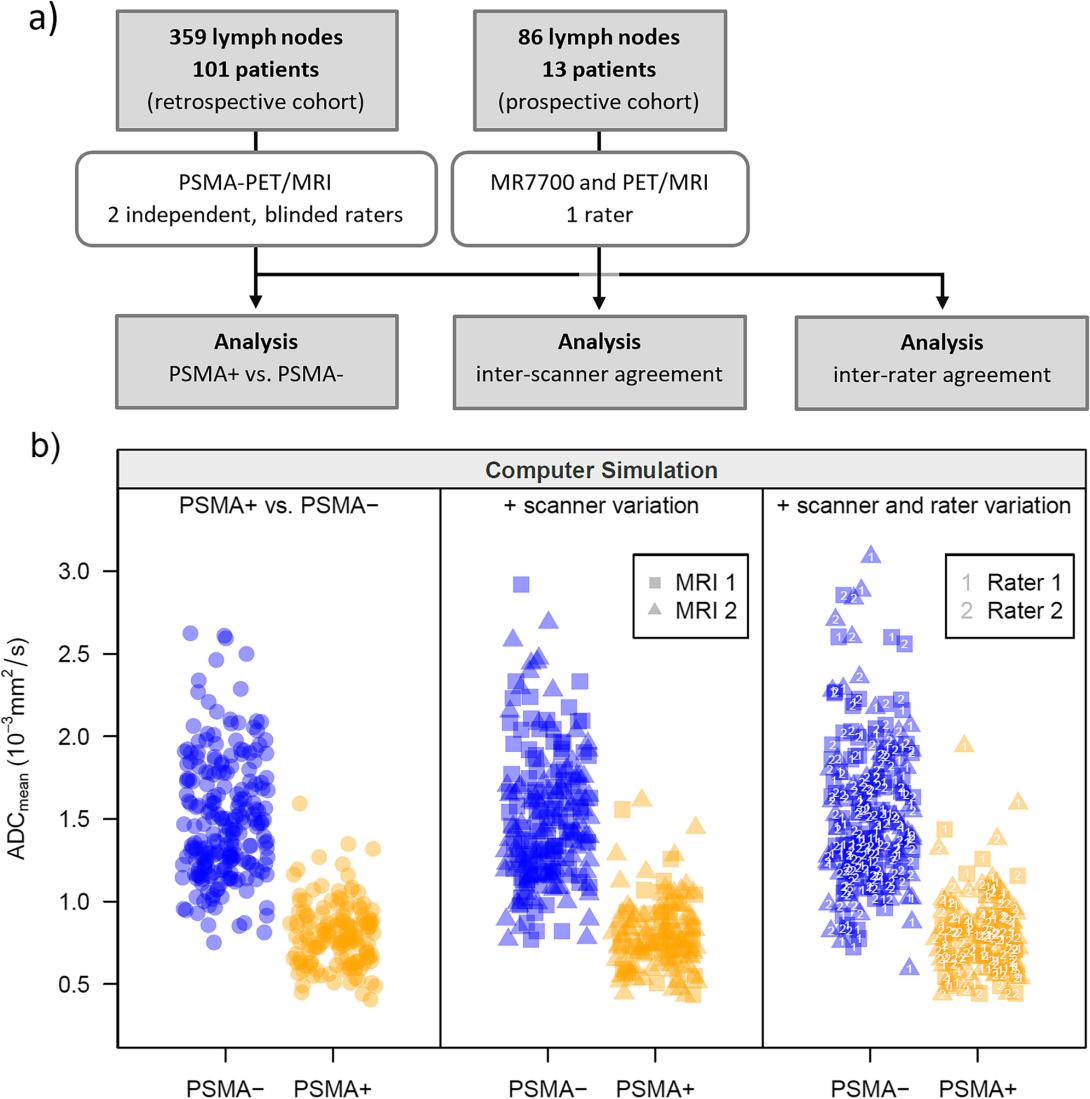
**a** Flow chart showing the number of patients in the two cohorts of prostate cancer patients. **b** Layout of the computer simulation study, symbolic representation of one simulation run for ADC_mean_

A cohort of 101 consecutive prostate cancer patients presenting for PSMA-PET/MRI on clinical grounds were retrospectively read to assess ADC values of benign and metastatic lymph nodes (Fig. 1a). Blinded readings of two readers were used to evaluate inter-rater variability. A second, prospective cohort of 13 prostate cancer patients was examined consecutively on two 3-T scanners to assess inter-scanner variability. This was complemented by a phantom study. Based on the analyses of inter-scanner and inter-rater agreement, a computer simulation study (Fig. 1b) was performed to assess the diagnostic accuracy of the ADC taking into account these two sources of variability.

### Phantom measurements

Phantom measurements were performed on a commercially available DWI phantom (https://qmri.com/) with an MR-readable thermometer to adjust target diffusion values for temperature (see also supplemental material).

### Imaging protocol

The two scanners used in this study were an MR7700 (Philips), hereinafter referred to as MRI 1, and a Biograph mMR (Siemens) (MRI 2). PSMA-PET was performed with 4 MBq/kg body weight [^68^Ga]Ga-PSMA-11 (*n﻿ *= 32) or [^18^F]F-PSMA-1007 (*n * = 69), injected 1 (^68^Ga) or 2 h (^18^F) prior to acquisition [18]. Patients were supine with arms down and MRI body coils attached. DIXON sequences were used for PET attenuation correction. PET scan was performed with 3 min per bed position from the skull to the tibia and simultaneous T1w and T2w MRI. DWI of the abdomen and pelvis followed after PET acquisition. PET image reconstruction was performed with onboard software using OSEM with 21 subsets and 3 iterations. See Table 1 for MRI sequence parameters.

**Table 1:** Protocol parameters for multiparametric MRI and comparison of the DWI-protocols

System	MRI 1: MR7700		MRI 2: mMR (PET/MRI)	
Sequence	T1-weighted (transaxial)	DWI (transaxial)	T1-weighted (transaxial)	DWI (transaxial)
Technique	TSE	SE-EPI	VIBE	SE-EPI
Repetition time/echo time (ms)	400–600/8	2200/84	4.07/1.96	6400/70
Flip angle (degrees)	90	90	9	90
No slices/slice thickness (mm)	31/5	21/5	48/5	21/5
Field of view (mm)	300 × 348	380 × 285	420 × 342	380 × 285
Acquisition matrix	300 × 348	192 × 143	320 × 224	97 × 160
Acquisition acceleration	Compressed SENSE 2	-	GRAPPA 2	GRAPPA 2
Fat suppression	-	SPIR	-	SPAIR
Acquisition time (min) (per bed position)	04:43	02:21	00:13	02:34
*b*-values (s/mm^2^)	-	0, 50, 400, 800	-	0, 50, 400, 800
Receiver-coil	16-ch-surface coil﻿ (dS anterior)		6-ch-surface coil (mMR body)	

Abbreviations: *DWI* diffusion-weighted imaging, *GRAPPA* generalized autocalibrating partial parallel acquisition, *SE-EPI* spin-echo-echoplanar imaging, *SENSE* sensitivity encoding, *SPAIR* spectral attenuated inversion recovery, *SPIR* spectral presaturation with inversion recovery, *TSE* turbo spin echo, *VIBE* volumetric interpolated breath/hold examination

### Image analysis

Centricity PACS RA1000 (General Electric) and syngo.via (Siemens Healthcare) were used for image display. The following lymph node stations were assessed: paraaortic/caval, common iliac, external iliac, obturator, internal iliac, presacral, pararectal, and inguinal [19].

PSMA-PET/MRI studies were viewed in an unblinded manner by one board-certified nuclear medicine specialist (B.N.) with 7 years of experience in hybrid imaging in a first step. All focal hyperintense structures on *b *= 50 s/mm^2^ DWI images were identified and correlated if they correspond to a lymph node on T1w images. All lymph nodes with a short-axis diameter ≥ 5 mm without a clear fatty hilus and discernible on ADC maps were noted. Lymph nodes were classified according to the EANM/SNMMI guideline as metastatic (PSMA+) when exhibiting tracer signal above the local background visually. All other were classified as non-metastatic (PSMA−) [18]. In a second step, ADC_mean_ and diameters of lymph nodes identified in the first step were measured independently and blinded to PSMA-PET by one radiologist (K.A.d.S.) and one nuclear medicine physician (W.R.) both with 6 years of experience in hybrid imaging. ADC_mean_ was measured with a polygonal region-of-interest placed on the ADC map following the lymph node shape as delineated on the *b *= 50 s/mm^2^ images.

MR image analysis for comparison of lymph node ADC measurements between the PET/MRI and MR7700 was conducted in a concordant manner by one reader (B.N.). No PET images were acquired for the smaller cohort.

### Statistical analysis

Normally distributed data are described using mean ± standard deviation, and non-normally distributed data using median and interquartile range (IQR, 25th–75th percentile).

The mean short-axis diameter and mean ADC_mean_ of the lymph node measurements of the two blinded raters were used to compare PSMA+ and PSMA− lymph nodes. To account for intra-individual correlation due to multiple lymph nodes per patient, linear mixed models (LMM) were applied. Both ADC_mean_ and short-axis diameter were log-transformed for all LMM analyses because of outliers or skewed distribution. Therefore, the mean estimated values must be understood as geometric means.

ROC curves and their corresponding area under the curve (AUC) were calculated to assess the diagnostic accuracy of ADC_mean_ and short-axis diameter on the lymph node level. The corresponding 95% confidence intervals (CI) and a comparison of the diagnostic value of both parameters were calculated, accounting for clustered data [20]. The cutoff maximizing Youden’s index was determined.

LMM-based intraclass correlation coefficients (ICC) were used to analyze the agreement of two raters or two scanners [21]. Additionally, Bland-Altman plots were generated depicting the bias (mean difference) ± limits of agreement (LoA) [22].

To analyze the impact of different sources of variation on diagnostic accuracy, a simulation study (10,000 runs per setting) based on the LMMs for inter-scanner and inter-rater agreement was performed. Each simulation run generated a dataset with a similar structure to the retrospective cohort or subset, but introduced additional variation by randomly selecting one rater and scanner for each patient (Fig. 1b).

Statistical analyses were performed using R version 4.2.0. Further details on the models and statistical analysis are provided in the supplement.

## Results

### Patient characteristics

The mean age of the 101 men undergoing simultaneous PSMA-PET/MRI was 68.3 ± 8.4 years (Table 2). The majority of patients presented for restaging in the setting of biochemical recurrent prostate cancer; another 4% of patients underwent PET/MRI for primary staging prior to prostatectomy. 62% of patients were without systemic therapy, while 38% received ongoing systemic therapy.

**Table 2:** Patient characteristics

		Retrospective cohort	Prospective cohort
Patients	*N*	101	13
Age (years)	Mean ± SD	68.3 ± 8.4	68.0 ± 6.0
PSA at study date (ng/mL)	Median (IQR)	2.5 (0.7–5.0)	0.8 (0.6–3.1)
Ongoing systemic therapy	Yes	38 (37.6%)	2 (15.4%)
	No	63 (62.4%)	11 (84.6%)
Number of lymph nodes	Median (IQR)	2 (1–4)	3 (2–9)
Lymph nodes	N	359	86
PSMA	PSMA+	143 (39.8%)	-
	PSMA−	216 (60.2%)	-
Short-axis diameter (mm)	Median (IQR)	7.0 (5.9–8.6)	6.0 (5.5–7.5)
Short-axis diameter	≤ 10 mm	302 (84.1%)	83 (96.5%)
	> 10 mm	57 (15.9%)	3 (3.5%)
Localization	Paraaortic/caval	77 (21.4%)	30 (34.9%)
	Common iliac	57 (15.9%)	18 (20.9%)
	External iliac/obturator	43 (12.0%)	11 (12.8%)
	Internal iliac	30 (8.4%)	5 (5.8%)
	Presacral/pararectal	26 (7.2%)	2 (2.3%)
	Inguinal	126 (35.1%)	20 (23.3%)

Abbreviations: *SD* standard deviation, *IQR* interquartile range

The second, smaller cohort of 13 men undergoing two consecutive MRI scans on different scanners was comparable concerning age (68.0 ± 6.0 years). Here, all patients presented for recurrent prostate cancer, with 85% of patients without systemic therapy, while 15% received ongoing systemic therapy.

### Lymph node characteristics

**Fig. 2:**
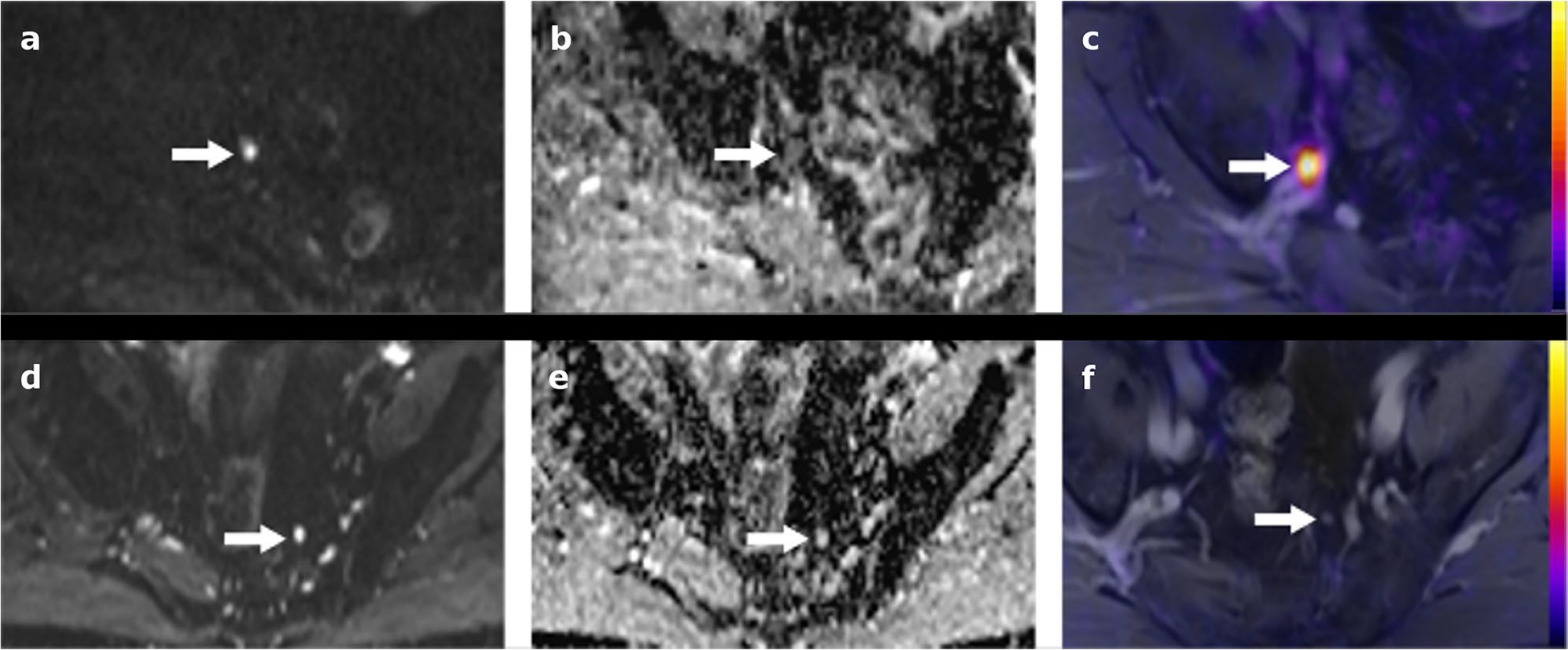
**a** Focal area of high signal intensity on *b*800 DWI image, corresponding to 6 × 7 mm large lymph node in T1w image (not shown), with low ADC value on the ADC map. **b** ADC_mean_ 0.81 × 10^−3^ mm^2^/s. **c** Fusion image of PSMA-PET and gadolinium-enhanced T1 showing strong, focal tracer signal (PSMA+). **d** In contrast, another focal area of high signal intensity on *b*50 DWI images, **e** corresponding to a 5 × 6 mm large lymph node, with comparably high ADC value on the ADC map, ADC_mean_ 1.26 × 10^−3^ mm^2^/s). **f** Fusion image of PSMA-PET and gadolinium-enhanced T1 image showing no apparent tracer signal (PSMA−)

In the patients undergoing PSMA-PET/MRI, 359 lymph nodes were detected in 101 patients based on DWI using the algorithm described above. Of those, 143 (39.8%) showed focal tracer signal on PSMA-PET (PSMA+) and were considered metastatic (Fig. 2), while 216 (60.2%) showed no signal on PSMA-PET (PSMA−) and were considered benign. The median short-axis diameter was 7 mm (IQR, 6–9; Table 2).

In the cohort of 13 patients for the comparison of MRI scanners, 86 lymph nodes with a median short-axis diameter of 6 mm (IQR, 6–8) were found.

### Difference between benign and malignant lymph nodes

**Fig. 3:**
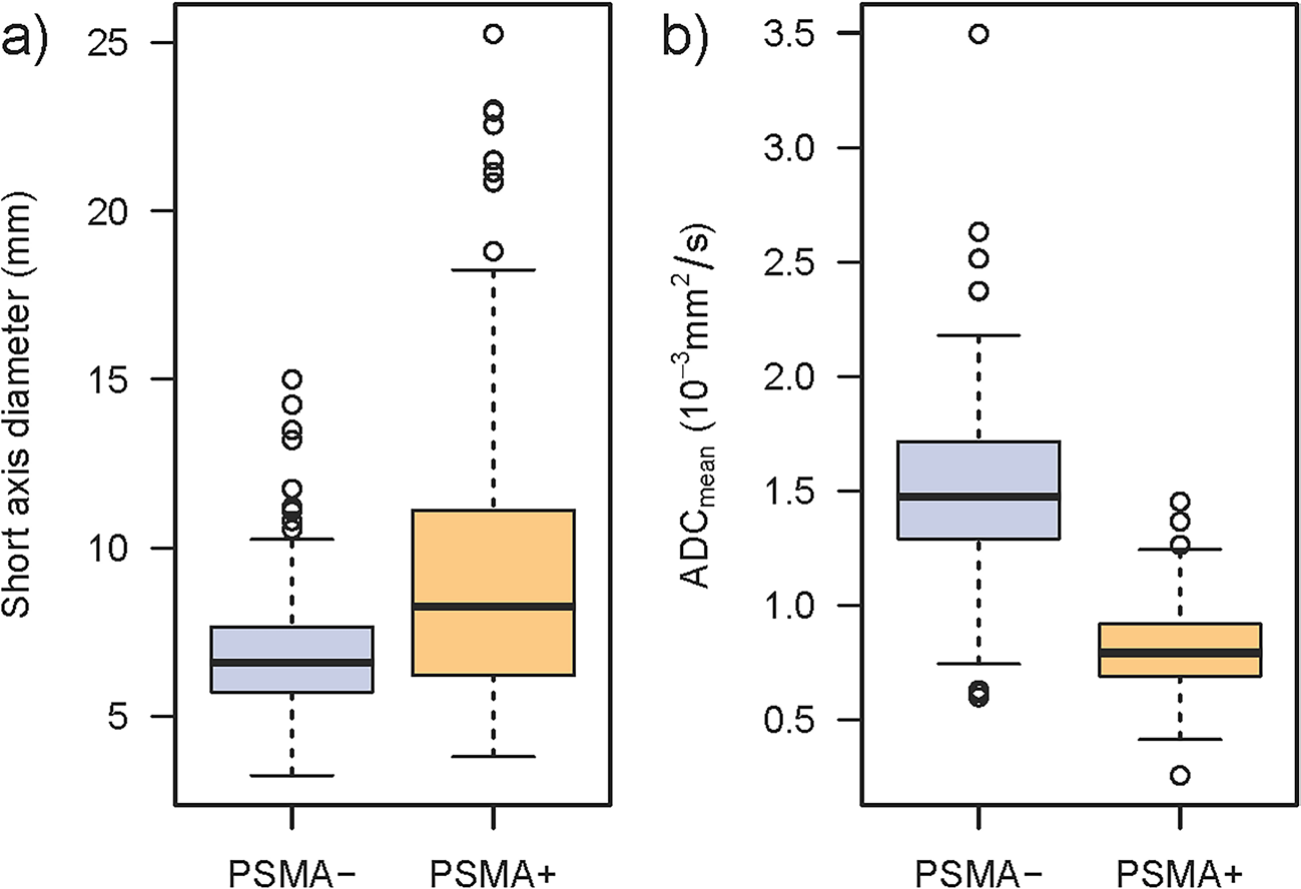
Boxplot showing the distribution of (**a**) short-axis diameter and (**b**) ADC_mean_ for lymph nodes with (PSMA+) and without tracer accumulation (PSMA−) in PSMA-PET

**Fig. 4:**
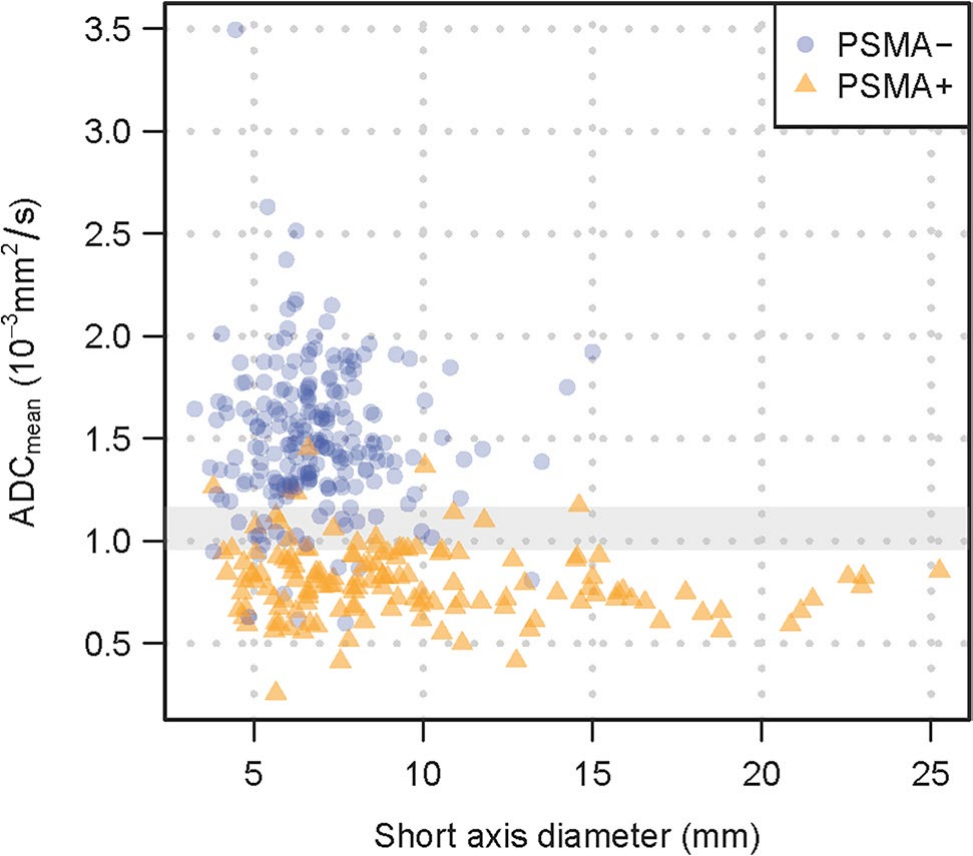
Scatterplot showing the discrimination of lymph nodes with (PSMA+) and without (PSMA−) tracer accumulation by ADC_mean_ and short-axis diameter. The ADC_mean_, in contrast to the short-axis diameter, allows a good differentiation between PSMA+ and PSMA− lymph nodes. The range of cutoffs for the ADC_mean_ resulting from the simulation study (2.5th–97.5th percentile) based on all lymph nodes is highlighted in gray

All measurements of the cohort used to analyze the differences in ADC values of benign and malignant lymph nodes were based on images taken with the PET/MRI scanner (101 patients, 359 lymph nodes). The mean short-axis diameter and mean ADC_mean_ measurements of the two blinded raters were used to compare PSMA+ and PSMA− nodes. Lymph nodes with PSMA signal showed a significantly lower ADC_mean_ (0.77 × 10^−3^ mm^2^/s) compared to lymph nodes without relevant PSMA signal (1.46 × 10^−3^ mm^2^/s, *p *< 0.001; Fig. 3, Table 3). The short-axis diameter was used as a comparison as the current quantitative standard. PSMA-positive lymph nodes presented with a greater short-axis diameter (8.3 mm) compared to PSMA-negative nodes (6.6 mm, *p *< 0.001; Fig. 3, Table 3). Unlike the short-axis diameter, the ADC_mean_ shows clear discrimination between PSMA-positive and PSMA-negative lymph nodes (Fig. 4).

**Table 3:** ADC_mean_ and short-axis diameter in PSMA+ and PSMA− lymph nodes estimated using linear mixed models. A total of 359 lymph nodes in 101 patients are included in the analysis including all lymph node regions. A total of 233 lymph nodes in 78 patients are included in the analysis excluding inguinal lymph nodes. A total of 302 lymph nodes in 99 patients are included in the analysis excluding lymph nodes > 10 mm

		PSMA−	PSMA+	Ratio PSMA+/PSMA−	*p-*value
All lymph node regions	Mean* ADC_mean_ (95%CI)	1.46 × 10^−3^ mm^2^/s (1.40–1.53)	0.77 × 10^−3^ mm^2^/s (0.73–0.81)	0.53 (0.50–0.56)	< 0.001
	Mean* short-axis diameter (95%CI)	6.6 mm (6.2–6.9)	8.3 mm (7.7–8.9)	1.26 (1.16–1.37)	< 0.001
Excluding inguinal lymph nodes	Mean* ADC_mean_ (95%CI)	1.43 × 10^−3^ mm^2^/s (1.33–1.53)	0.78 ×10^−3^ mm^2^/s (0.74–0.83)	0.55 (0.50–0.59)	< 0.001
	Mean* short-axis diameter (95%CI)	6.6 mm (6.0–7.3)	8.1 mm (7.4–8.8)	1.23 (1.09–1.39)	0.001
Excluding lymph nodes > 10mm	Mean* ADC_mean_ (95%CI)	1.47 × 10^−3^ mm^2^/s (1.41–1.53)	0.79 × 10^−3^ mm^2^/s (0.74–0.83)	0.54 (0.50–0.57)	< 0.001
	Mean* short-axis diameter (95%CI)	6.4 mm (6.2–6.6)	6.7 mm (6.4, 7.1)	1.05 (0.99, 1.11)	0.1

^*^Geometric mean

Since prostate cancer metastases to inguinal lymph nodes are rare and the maximum short-axis diameter in inguinal lymph nodes is larger than in retroperitoneal lymph nodes [23, 24], the analysis was repeated excluding inguinal lymph nodes with comparable results (Table 3).

ROC analysis resulted in a markedly larger AUC for ADC_mean_ (0.97, Table 4) than for short-axis diameter (0.69, *p *< 0.001) (Figure S1). The cutoff for ADC_mean_ was calculated at 1.02 × 10^−3^ mm^2^/s with a sensitivity of 92% and a specificity of 94% on a lymph node basis. For short-axis diameter, the cutoff was 8.6 mm with a sensitivity of 48% and a specificity of 89%. Again, results were comparable when excluding inguinal lymph nodes (Table 4). Limiting the ROC analysis to lymph nodes inconspicuous regarding size (i.e. ≤ 10 mm) led to an AUC of 0.97 for ADC_mean_ (Table 4).

**Table 4:** Diagnostic accuracy of ADC_mean_ and short-axis diameter to distinguish between PSMA+ and PSMA− lymph nodes. A total of 359 lymph nodes in 101 patients are included in the analysis including all lymph node regions. A total of 233 lymph nodes in 78 patients are included in the analysis excluding inguinal lymph nodes. A total of 302 lymph nodes in 99 patients are included in the analysis excluding lymph nodes > 10 mm

		ADC_mean_	Short-axis diameter
All lymph node regions	Area under curve (95% CI)	0.967 (0.942, 0.993)	0.686 (0.600, 0.771)
	Cutoff maximizing Youden’s index	1.02 × 10^−3^ mm^2^/s Sensitivity: 92% (131/143) Specificity: 94% (204/216)	8.6 mm Sensitivity: 48% (69/143) Specificity: 89% (192/216)
	Difference in AUC (95% CI)	0.282 (0.195, 0.368) *p* < 0.001	
Excluding inguinal lymph nodes	Area under curve (95% CI)	0.947 (0.896, 0.999)	0.682 (0.596, 0.768)
	Cutoff maximizing Youden’s index	1.01 × 10^−3^ mm^2^/s Sensitivity: 91% (127/140) Specificity: 94% (87/93)	8.6 mm Sensitivity: 46% (65/140) Specificity: 88% (82/93)
	Difference in AUC (95% CI)	0.266 (0.172, 0.360) *p* < 0.001	
Excluding lymph nodes > 10mm	Area under curve (95% CI)	0.968 (0.944, 0.991)	0.572 (0.482, 0.661)
	Cutoff maximizing Youden’s index	1.09 × 10^−3^ mm^2^/s Sensitivity: 95% (92/97) Specificity: 92% (188/205)	7.9 mm Sensitivity: 37% (36/97) Specificity: 84% (172/205)
	Difference in AUC (95% CI)	0.396 (0.303, 0.489) *p* < 0.001	

### Inter-rater reliability

The ADC_mean_ measurements of the two blinded raters of 359 lymph nodes in 101 patients that underwent PSMA-PET/MRI showed excellent inter-rater agreement (ICC 0.93, 95%CI [0.92, 0.95]). Only a small bias was observed between the two raters (mean difference 0.013 × 10^−3^ mm^2^/s; Figure S2). The limits of agreement are ± 0.29 × 10^−3^ mm^2^/s from the mean difference of the two raters.

Results for the short-axis diameter are given in the supplement (Figure S3).

### Inter-scanner reliability

High agreement of the two scanners was observed concerning ADC_mean_ in the phantom study (ICC 0.999, 95%CI [0.995, 1]). Further results of the phantom study are presented in the supplement (Figures S4 and S5, Table S1).

Agreement of the two scanners was then tested on the smaller cohort (13 patients, 86 lymph nodes). In this case, all lymph nodes were measured by the same rater. Only a small bias was found for the ADC_mean_ (mean difference 0.002 × 10^−3^ mm^2^/s; Figure S6). The limits of agreement were ± 0.35 × 10^−3^ mm^2^/s (or 29.2% when calculating the LoAs for the difference divided by the mean). Overall, the measurements of the two scanners agreed well with an ICC of 0.89 (95%CI [0.84, 0.93]). Comparing these results with the close agreement observed in the phantom measurements shows that the difference of measurements in the patient cohort is not only caused by inter-scanner variation but also by lower repeatability of in vivo measurements.

Results for the short-axis diameter are given in the supplement (Figure S7).

### Computer simulation study of robustness of ADC-based lymph node classification

**Fig. 5:**
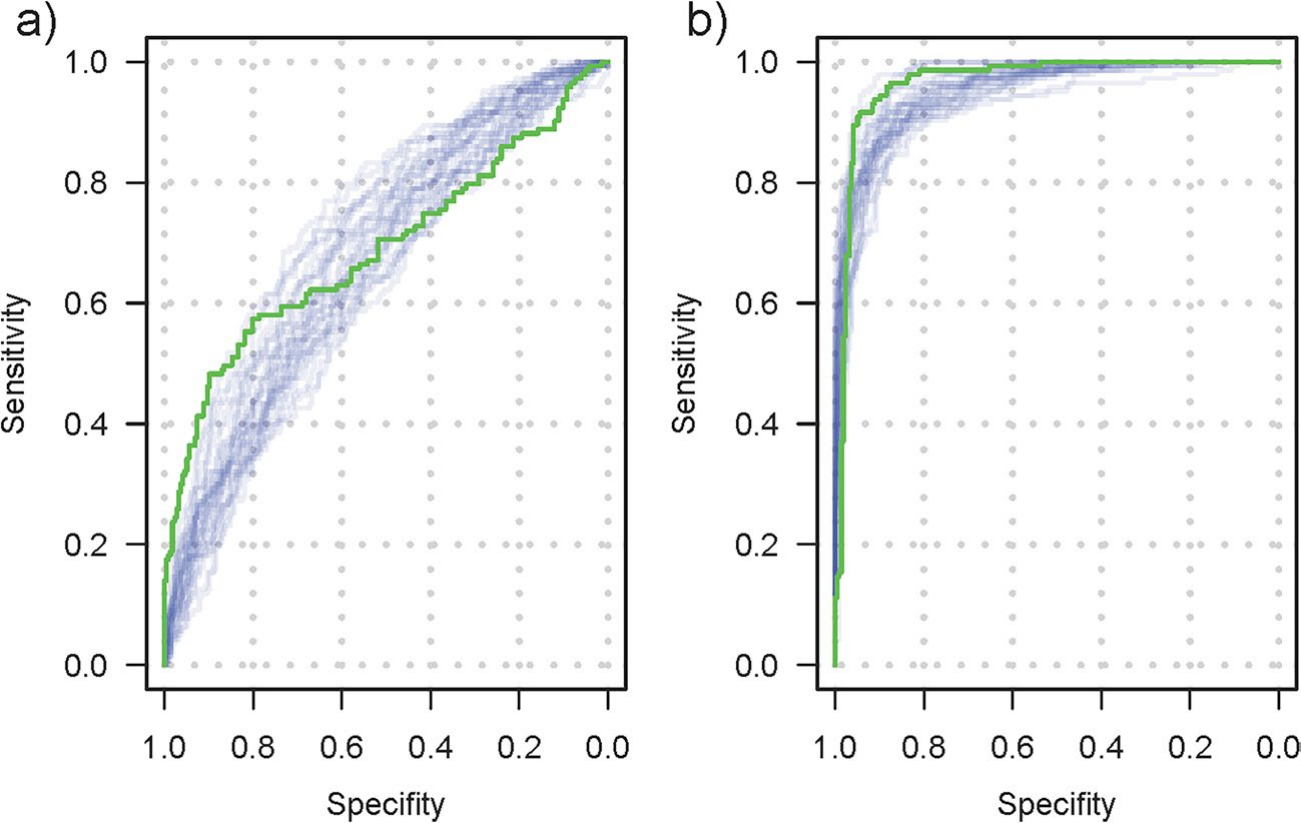
ROC curves generated by 30 exemplary simulation runs based on the datasets including all lymph nodes for diagnostic accuracy concerning PSMA−/+ considering inter-scanner and inter-rater variability. The opaque green curves are the ROC curves observed for the average measurements made by two blinded raters. **a** Simulated ROC curves for short-axis diameter. **b** Simulated ROC curves for ADC_mean_

**Fig. 6:**
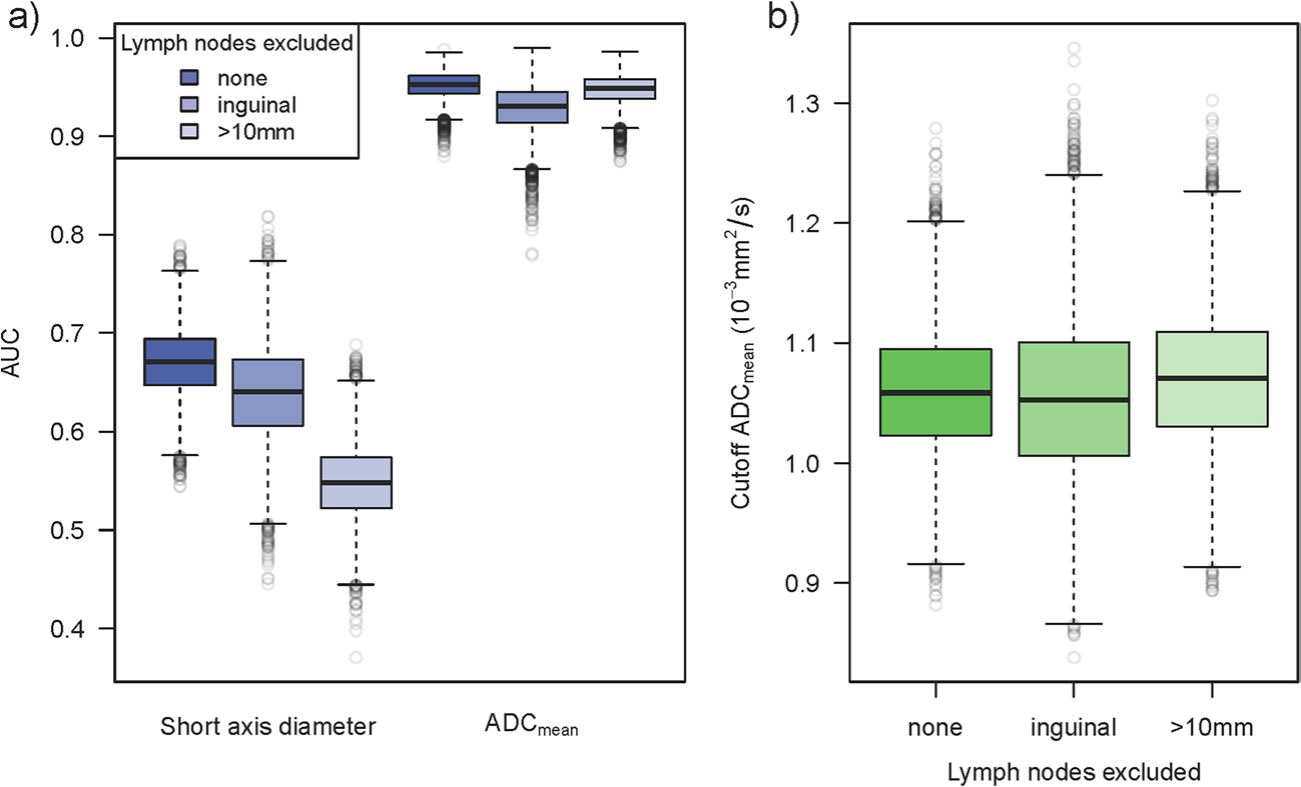
Results of the simulation study accounting for inter-scanner and inter-rater variability (10,000 simulation runs per setting). The simulations were based on the full datasets including all lymph nodes (359 lymph nodes in 101 patients), the datasets excluding inguinal lymph nodes (233 lymph nodes in 78 patients), and the datasets excluding lymph nodes > 10 mm (302 lymph nodes in 99 patients). **a** AUC of the simulated ROC curves for short-axis diameter and ADC_mean_. **b** Cutoffs maximizing Youden’s index resulting from the simulated ROC curves for ADC_mean_

A computer simulation was programmed to evaluate the impact of inter-rater and inter-scanner variability on diagnostic accuracy. In each simulation run (10,000 runs per setting), a dataset was created that was similar in size and structure to the original retrospective dataset or a subset. The previous results on the difference of the ADC value depending on the status were used to simulate measurements of PSMA+ and PSMA− lymph nodes. Results on inter-rater and inter-scanner variance were used to mimic the blurring that occurs in the real-world application due to differing scans, scanners, and raters (Fig. 1b). A ROC analysis was performed for each simulated data set (Fig. 5). The results show that the diagnostic accuracy for ADC_mean_ decreases when considering the additional sources of variation but is still very good with a median AUC of 0.95 (95%CI [0.92, 0.97]; Fig. 6a, Table S2). The classification capacity is maintained when excluding inguinal lymph nodes or lymph nodes > 10 mm. Also, the cutoff maximizing Youden’s index was calculated in each simulation run, resulting in a 2.5th and 97.5th percentile of 0.96 × 10^−3^ and 1.17 × 10^−3^ mm^2^/s, respectively, for the full cohort (Figs. 4 and 6b). The median AUC for the short-axis diameter resulting from the simulation based on the full dataset is 0.67 (95%CI [0.60, 0.74]; Fig. 6a). Although residual analysis showed a good model fit, the shape of the simulated ROC curves differs from the observed curve. Nevertheless, the results illustrate that the short-axis diameter is inferior to the ADC_mean_ in diagnostic accuracy.

## Discussion

This study evaluated the robustness of ADC-based lymph node classification in the presence of both inter-rater and inter-scanner variability. ADC values were substantially lower in metastatic lymph nodes, resulting in a high diagnostic accuracy even when considering the variability introduced by different raters and scanners.

Previous studies have yielded conflicting results on whether metastatic and benign lymph nodes in prostate cancer differ in ADC. Four studies totaling about 100 patients found statistically significant differences in ADC values [11–14], while two similarly powered studies found lower but not statistically significant different ADC values for malignant nodes [15, 16]. Our cohort of 101 patients supports the hypothesis that metastatic and benign lymph nodes differ in their ADC values. Also, the ADC values we observe in benign lymph nodes are in line with the findings of Donners et al [25]. ROC analysis demonstrated excellent diagnostic performance of the ADC_mean_ for lymph node classification with an AUC of 0.97, corroborating preceding studies [11, 12, 14]. However, even with significant differences between malignant and benign lymph nodes, the diagnostic capacity might be obscured in practice due to inter-scanner and inter-rater variability.

Overall, we found a high reliability of ADC measurements with an excellent inter-rater reliability for ADC measurements (ICC 0.93, 95%CI [0.92, 0.95]). These findings are well in line with Heijnen et al, investigating 102 pelvic lymph nodes in 21 rectal cancer patients reporting an ICC of 0.86 [26]. Smaller, previous studies in other lymph node regions reported a comparable agreement [27, 28].

Concerning inter-scanner reliability, the phantom study showed good measurement precision, well in line with previous results [28–31]. A high agreement between the scanners could also be demonstrated for in vivo measurements of lymph nodes (ICC 0.89, 95%CI [0.84, 0.93]), notably, without complete harmonization of sequences or post-processing software. Bland–Altman analysis demonstrated only a slight bias between the two scanners of 0.002 × 10^−3^ mm^2^/s (LoA ± 0.35 × 10^−3^ mm^2^/s or 29.2%). To our knowledge, there are only a few studies investigating the reproducibility of ADC measurements in human subjects, which are, however, well in line with our results. Hoang-Dinh et al investigating ADC reproducibility in normal prostate peripheral zones report a bias between two scanners of 0.01 × 10^−3^ mm^2^/s (LoA ± 0.23 × 10^−3^ mm^2^/s) [32]. Michoux et al investigating reproducibility in healthy volunteers report LoAs ranging from ± 9% in white matter to ± 31% in the spleen besides higher values in osseous tissues [31].

So far, classification capacity and measurement variability of ADC have only been considered independently and not holistically. Addressing this gap, we conducted a computer simulation study evaluating the impact of scanner and rater variance. Even in the presence of additional sources of variability, the excellent classification capacity of ADC is maintained (AUC 0.95). The cutoff value of 1.02 × 10^−3^ mm^2^/s for ADC, found in our study, and the simulated cutoff range from 0.96 to 1.17 × 10^−3^ mm^2^/s are well in line with previously reported cutoffs ranging from 0.91 × 10^−3^ to 1.43 × 10^−3^ mm^2^/s [12, 14].

Traditionally, the cutoff for identifying suspicious lymph nodes by size is a short-axis diameter of 8–10 mm [24, 33], which is corroborated by our data. Moreover, the simulated AUC of only 0.67 confirms the limited diagnostic capacity of lymph node size found in previous studies [11, 12]. When considering only lymph nodes ≤ 10 mm, nodule size virtually loses its classification capacity (AUC 0.55). In contrast, ADC retains a high classification capacity for small lymph nodes, even when additional sources of variability are taken into account.

### Limitations

Our study has several limitations. For one, PSMA-PET is a good but not perfect standard of reference. It offers a very high, but inherently not perfect accuracy, among others, due to limited spatial resolution, metastases not over-expressing PSMA, or benign processes resulting in PSMA over-expression [34, 35]. However, in this specific scenario, PSMA-PET offers advantages compared to histology. Lymph nodes are numerous and often small, making it virtually impossible to match all nodes between imaging and histology. Therefore, some previous studies analyzed on the level of nodal stations rather than on individual nodes, resulting in averaging of ADC values [14, 15]. Other studies limited the analysis to a subset of possibly non-representative lymph nodes which might narrow the difference between malignant and benign nodes [13, 16]. In our study, patients were examined in a dedicated PET/MRI scanner, assuring spatial co-registration of simultaneously acquired PET and MRI. Using PSMA-PET as a reference standard, we could compare ADC values on node-by-node level rather than on a lymph station level.

Another limitation is that we only compared two raters and two scanners, so we cannot make general statements about the variability caused by raters, scanners, or technical parameters. Also, all measurements used for comparing lymph nodes with and without PSMA signal were made with the same scanner. However, the level of agreement between scanners and raters observed in our study as well as by others and the simulation suggest that the results are transferable.

## Conclusion

In conclusion, ADC values of benign and metastatic lymph nodes in prostate cancer differ significantly, resulting in a high classification capacity of the ADC that is robust to the variability arising from different raters and scanners.

### Supplementary Information

Below is the link to the electronic supplementary material.

Supplementary file1 (DOCX 1.62 MB)

## Abbreviations

ADC: Apparent diffusion coefficient
AUC: Area under the curve
CI: Confidence interval
DWI: Diffusion-weighted magnetic resonance imaging
ICC: Intraclass correlation coefficient
IQR: Interquartile range
LMM: Linear mixed model
LoA: Limits of agreement
MRI: Magnetic resonance imaging
PET: Positron emission tomography
PSMA: Prostate-specific membrane antigen
ROC: Receiver operating characteristic

## Declarations

### Guarantor

The scientific guarantor of this publication is Benjamin Noto.

### Statistics and biometry

One of the authors has significant statistical expertise, as Maria Eveslage has a degree in statistics.

### Study subjects or cohorts overlap

Parts of the results were submitted as an abstract to the 2023 ISMRM & ISMRT Annual Meeting.

### Methodology

• prospective and retrospective

• diagnostic or prognostic study

• performed at one institution
