# Supplementary material for: Robustness of apparent diffusion coefficient–based lymph node classification for diagnosis of prostate cancer metastasis
Source: Eur Radiol. 2023 Dec 15;34(7):4504–15. doi: 10.1007/s00330-023-10406-8 (PMC11213742; doi:10.1007/s00330-023-10406-8)
Supplement: Supplementary file 1 — Supplementary file1 (DOCX 1.62 MB) [file 330_2023_10406_MOESM1_ESM.docx]

**Supplemental Material**

**Robustness of ADC-based Lymph Node Classification for Diagnosis of Prostate Cancer Metastasis**

**Statistical analysis**

To account for intra-individual correlation due to multiple lymph nodes per patient in the comparison of PSMA+ and PSMA- lymph nodes concerning mean ADC_mean_ and mean short axis diameter, linear mixed models (LMM) [1] were used. The models included PSMA classification (+/-) as fixed effect and a random intercept for the patient. The model fit of LMMs was assessed descriptively.

To analyze the agreement of the two blinded raters as well as the two scanners on ADC_mean_ or short axis diameter in in-vivo measurements, ICCs based on the estimated variance components of LMMs were calculated. The LMMs included a random intercept each for the patient and the lymph node within the patient and a fixed effect for the rater or the scanner [2]. A confidence interval for the ICC was determined using a parametric bootstrap (10,000 runs) [3; 4].

In the Bland-Altman analysis [5], the limits of agreement (LoA) were calculated with and without adjusting for the fact that in many patients more than one lymph node was measured [6]. As the correlation of the differences between the measurements of the two raters or scanners within the patients was negligible (i.e., constant random variation between raters or scanners across patients), values not adjusted for repeated measurements are presented.

For ADC measurements made using the phantom, mean measured ADC_mean_ as well as the mean and maximum deviation from the target value of the respective concentration were calculated. Measurements were additionally evaluated with linear regression, as no relevant correlation was observed within the measurement runs. The scanner, the concentration-dependent target value, and the measurement run number were used as fixed effects to calculate the mean difference between the scanners. To assess the agreement of the two scanners, a LMM based ICC with scanner as fixed effect and concentration as random effect was calculated [2].

For the simulation study, a parametric bootstrap based on the LMMs fit for the analysis of inter-scanner as well as inter-rater agreement was used. Therefore, the estimated regression coefficients and variance components from the mixed model for the comparison of the two raters as well as PSMA+ and PSMA- lymph nodes (retrospective cohort) were used as a basis. The systematic and random error from the model for the comparison of the two scanners (prospective cohort) were added. In each simulation run, one of the raters and one of the scanners were randomly selected for all lymph nodes of a simulated patient. All models were fit using R version 4.2.0 [7] using lme4 version 1.1-29 [8].

**Figure S1:** ROC curves for the diagnosis of PSMA+ lymph nodes using ADC_mean_ and short axis diameter. The analysis is based on measurements of two blinded raters averaged by lymph node for 359 lymph nodes in 101 patients.


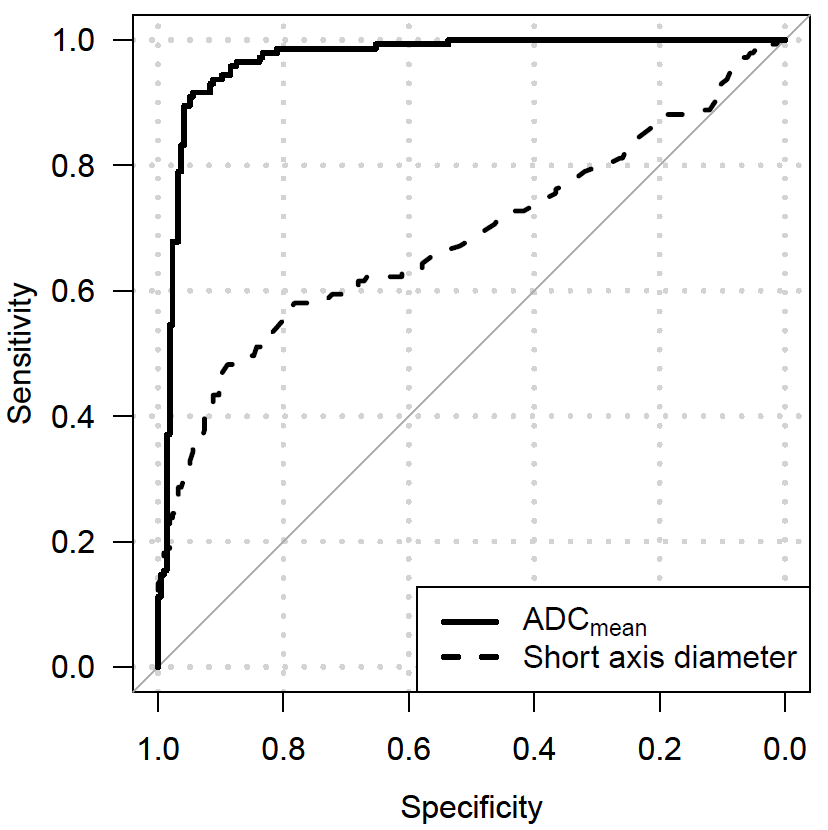


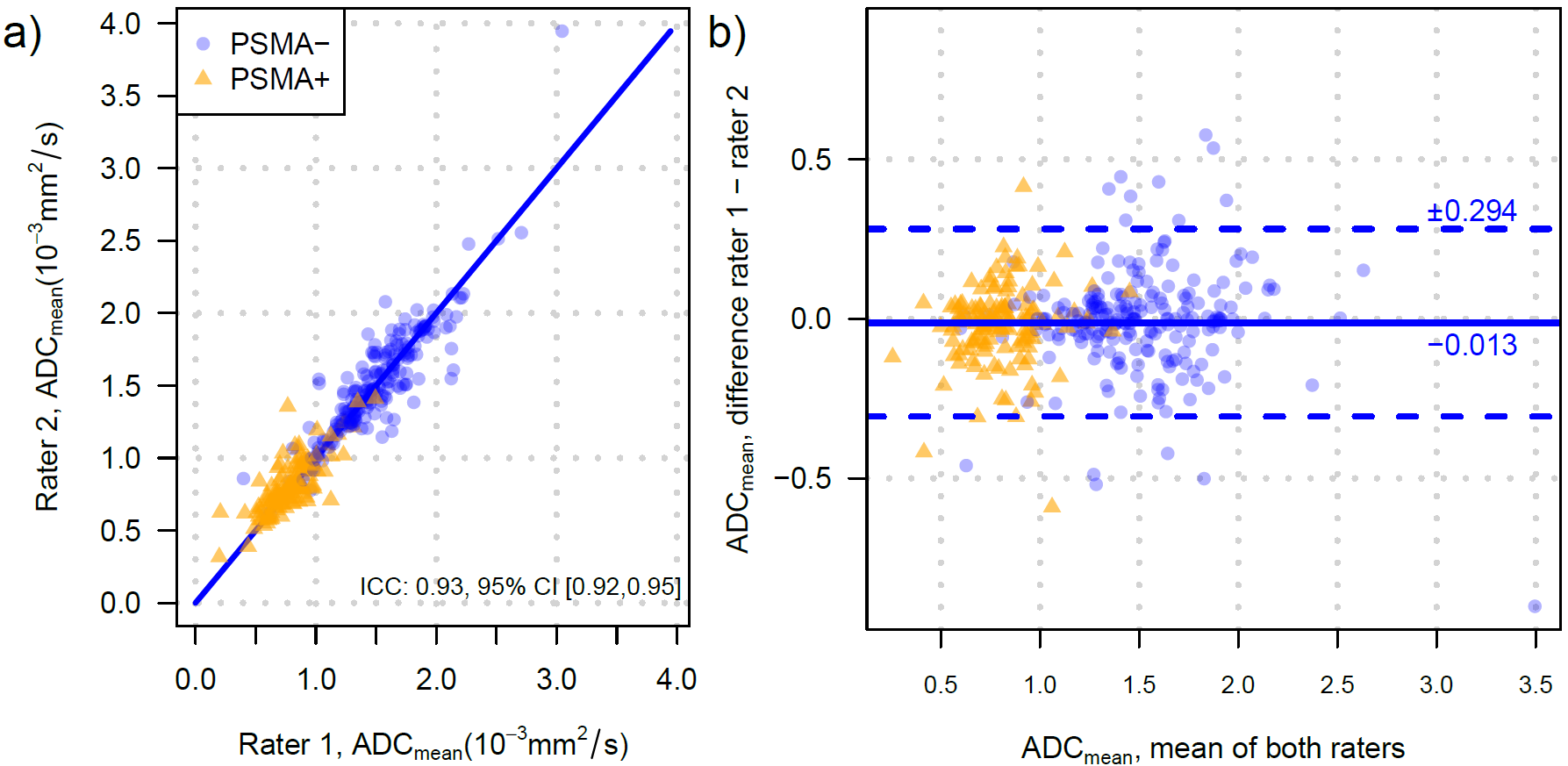


**Figure S2**: a) Agreement of the measurement of the ADC_mean_ (10^-3^mm²/s) of the two raters in 359 lymph nodes (101 patients). The solid diagonal line indicates perfect agreement. The intraclass correlation coefficient (ICC) is given in the figure. b) Bland-Altman-plot for the measurement of the ADC_mean_ (10^-3^mm²/s) of the two raters. The solid line represents the mean difference and the dashed lines the limits of agreement. The mean difference and the distance of the limits of agreement from the mean difference are indicated.


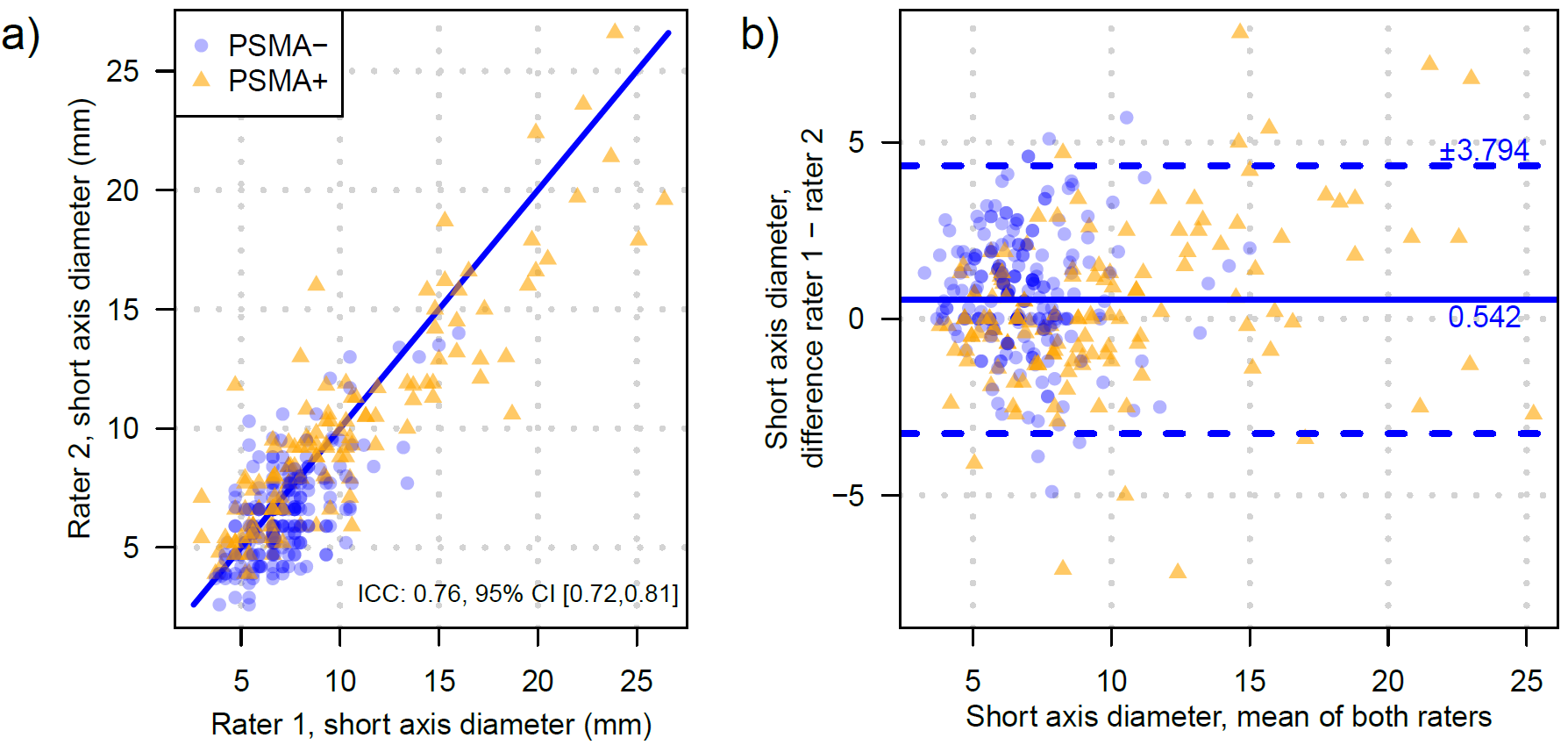


**Figure S3**: a) Agreement of the measurement of the short axis diameter (mm) of the two raters in 359 lymph nodes (101 patients). The solid diagonal line indicates perfect agreement. The intraclass correlation coefficient (ICC) is given in the figure. b) Bland-Altman-plot for the measurement of the short axis diameter (mm) of the two raters. The solid line represents the mean difference and the dashed lines the limits of agreement. The mean difference and the distance of the limits of agreement from the mean difference are indicated.

**Phantom measurements**

The DWI phantom contains two vials each with 10%, 20%, 30%, 40%, and 50% concentration of polyvinylpyrrolidone and three vials with 0%. Five measurement runs were performed in the isocenter of each scanner with the same sequences and coils used for the patient study (Table 1). All measurements per scanner were performed on the same day.


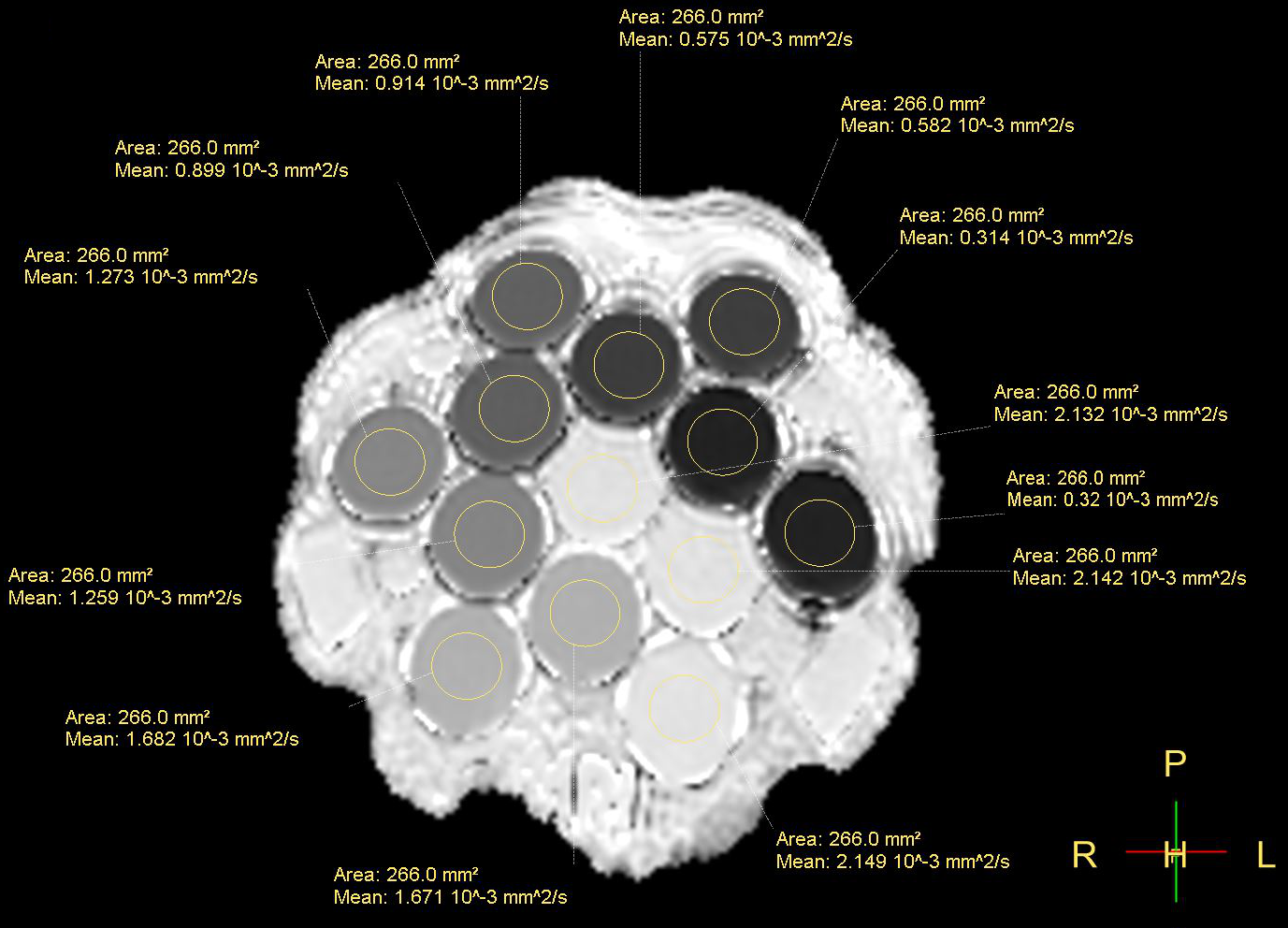


**Figure S4**: Exemplary phantom measurement on the Philips MR7700.

**Table S1**: Results of ADC measurements using the phantom. Measurements of PET/MRI were on average 0.016x10^-3^mm²/s (95%CI [0.010, 0.021], p < 0.001) higher than those from the MR7700. (PVP: polyvinylpyrrolidone, SD: standard deviation)

|  | | | ADC_mean_ (10^-3^mm²/s) | | | | | | |
| --- | --- | --- | --- | --- | --- | --- | --- | --- | --- |
|  |  |  | **MRI 1: MR7700** | | | **MRI 2: PET/MRI** | | | |
| PVP concentration | Number of measurements per scanner | Temperature-adjusted ADC_mean_ target value  (10^-3^mm²/s) | Mean ± SD | Absolute deviation from target value,  mean ± SD | Maximum absolute deviation from target value  (%) | | Mean ± SD | Absolute deviation from target value,  mean ± SD | Maximum absolute deviation from target value  (%) |
| 50% | 5x2 | 0.293 | 0.320  ± 0.007 | 0.027  ± 0.007 | 0.038 (13.0) | | 0.336  ± 0.005 | 0.043  ± 0.005 | 0.051 (17.4) |
| 40% | 5x2 | 0.545 | 0.577  ± 0.007 | 0.032  ± 0.007 | 0.040 (7.3) | | 0.600  ± 0.008 | 0.055  ± 0.008 | 0.070 (12.8) |
| 30% | 5x2 | 0.886 | 0.905  ± 0.010 | 0.019  ± 0.010 | 0.032 (3.6) | | 0.912  ± 0.004 | 0.026  ± 0.004 | 0.034 (3.8) |
| 20% | 5x2 | 1.258 | 1.269  ± 0.012 | 0.014  ± 0.008 | 0.025 (2.0) | | 1.275  ± 0.003 | 0.017  ± 0.003 | 0.024 (1.9) |
| 10% | 5x2 | 1.640 | 1.680  ± 0.012 | 0.040  ± 0.012 | 0.054 (3.3) | | 1.693  ± 0.005 | 0.053  ± 0.005 | 0.061 (3.7) |
| 0% | 5x3 | 2.106 | 2.148  ± 0.014 | 0.042  ± 0.014 | 0.058 (2.8) | | 2.172 ± 0.013 | 0.066  ± 0.013 | 0.084 (4.0) |


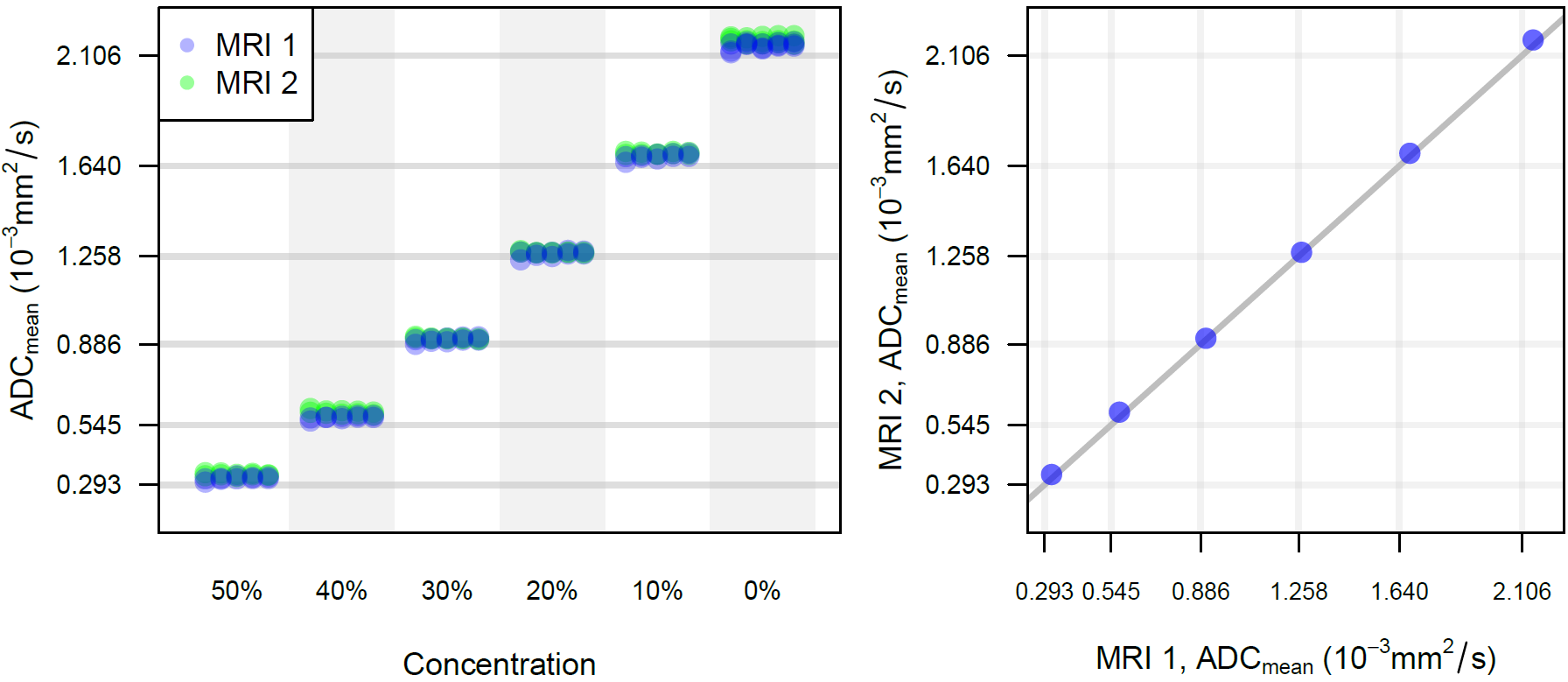


**Figure S5**: a) ADC measurements using the phantom. Five measurement runs were made with each scanner and the resulting measured values per run are arranged in chronological order from left to right at the respective concentration. The horizontal lines indicate the ADC_mean_ target values. b) Agreement of the ADC measurements of the two scanners using the phantom. All readings per concentration and scanner were averaged. Horizontal and vertical lines indicate the ADC_mean_ target values. The diagonal line indicates perfect agreement.


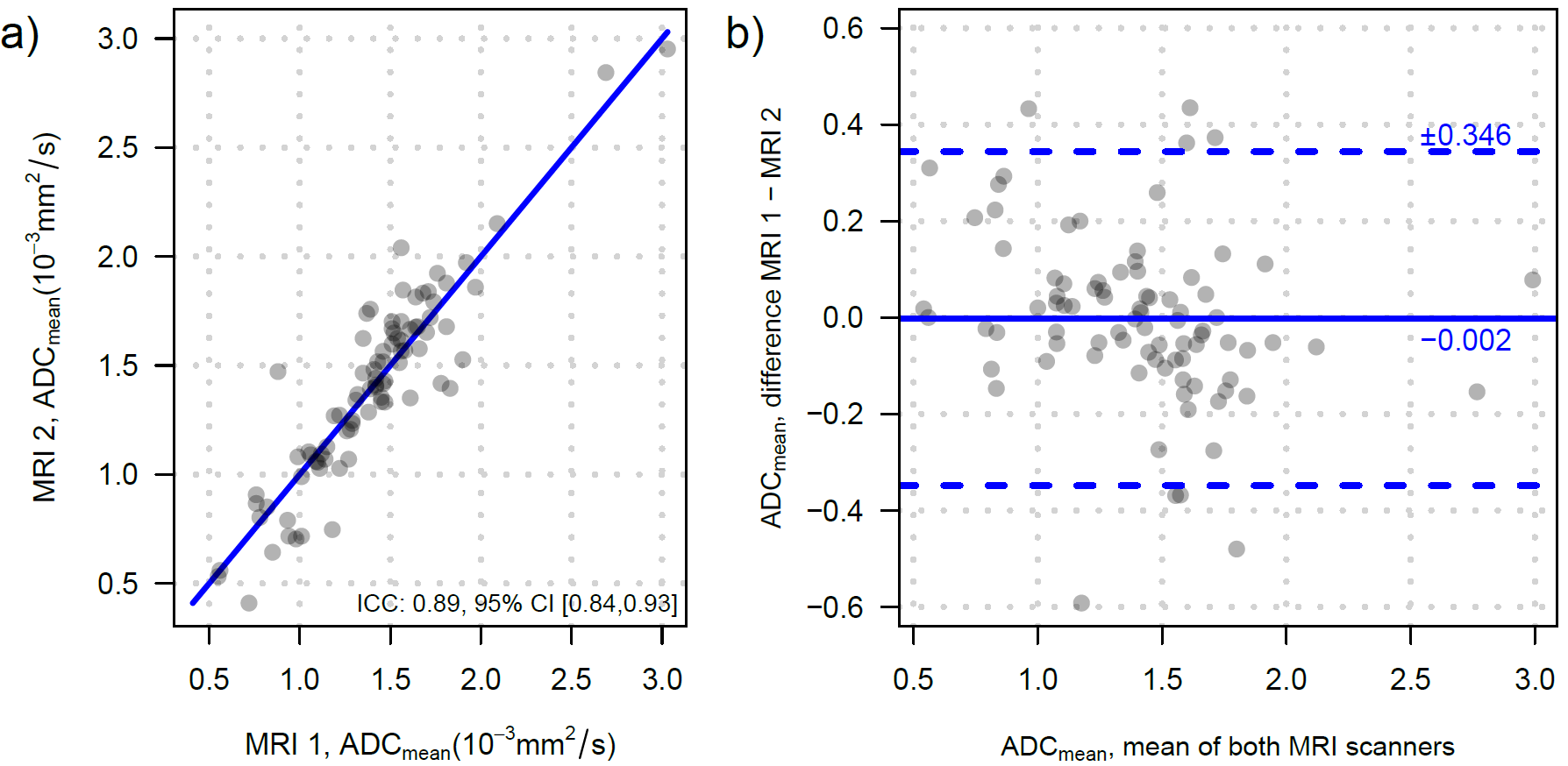


**Figure S6**: a) Agreement of the measurement of the ADC_mean_ (10^-3^mm²/s) of the two scanners in 86 lymph nodes (13 patients). The solid diagonal line indicates perfect agreement. The intraclass correlation coefficient (ICC) is given in the figure. b) Bland-Altman-plot for the measurement of the ADC_mean_ (10^-3^mm²/s) of the two scanners. The solid line represents the mean difference and the dashed lines the limits of agreement. The mean difference and the distance of the limits of agreement from the mean difference are indicated.


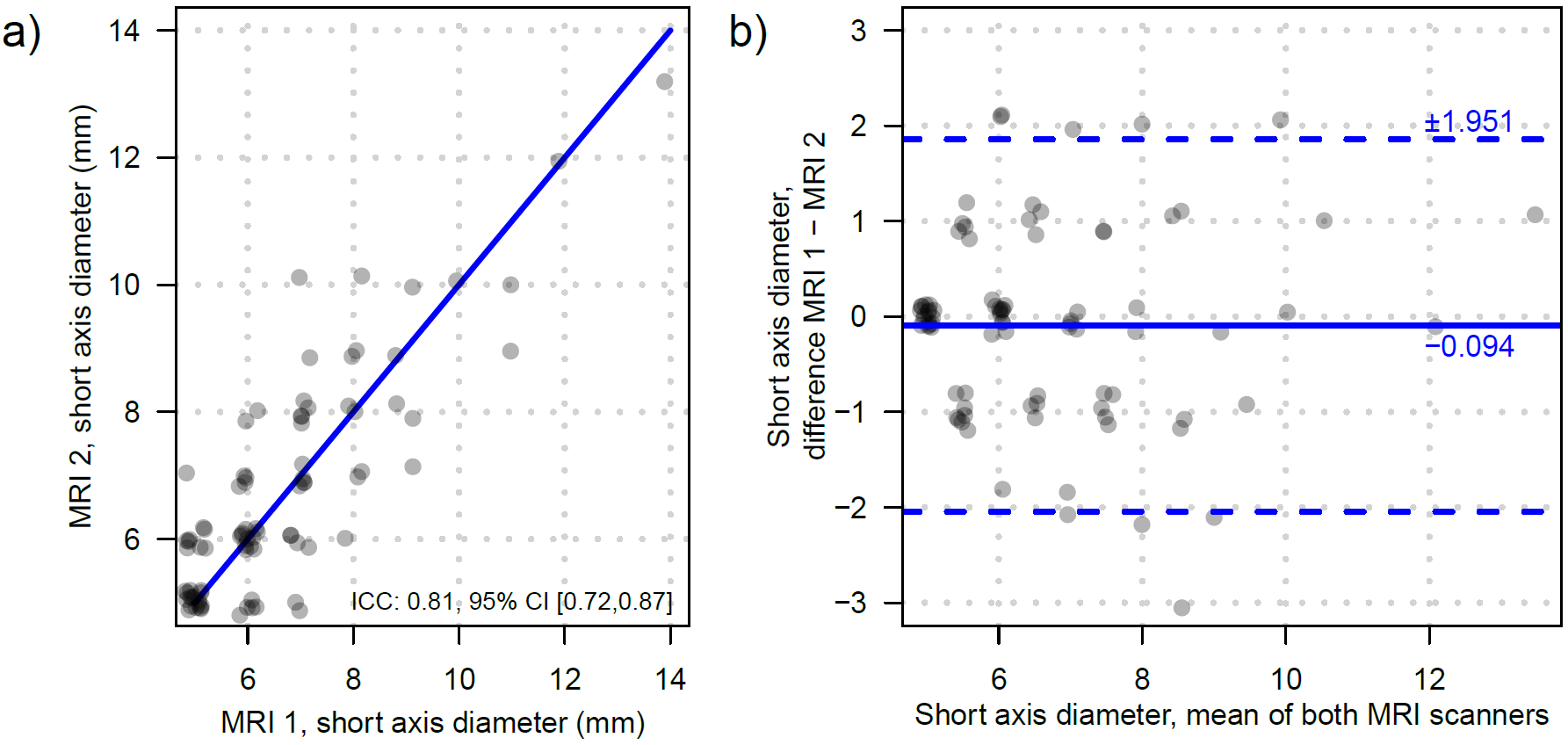


**Figure S7**: a) Agreement of the measurement of the short axis diameter (mm) of the two scanners in 86 lymph nodes (13 patients). The solid diagonal line indicates perfect agreement. The intraclass correlation coefficient (ICC) is given in the figure. b) Bland-Altman-plot for the measurement of the short axis diameter (mm) of the two scanners. The solid line represents the mean difference and the dashed lines the limits of agreement. The mean difference and the distance of the limits of agreement from the mean difference are indicated.

**Please note:** Small random errors have been added in a) and b) to make overlapping points more visible (for plotting purposes only). The function jitter was used resulting in a random error, that is uniformly distributed with mean 0 and variance equal to one seventy-fifth of the squared smallest difference of unique measured values.

**Table S2**: Results of the simulation study (10,000 simulations runs per setting).

|  | | **Subset** | | |
| --- | --- | --- | --- | --- |
|  |  | **All lymph nodes** | **Excluding inguinal lymph nodes** | **Excluding lymph nodes > 10mm** |
| **Size of datasets used as basis for simulation** | Dataset on rater agreement | 359 lymph nodes 101 patients | 233 lymph nodes 78 patients | 302 lymph nodes 99 patients |
|  | Dataset on scanner agreement | 86 lymph nodes 13 patients | 66 lymph nodes 12 patients | 83 lymph nodes 13 patients |
| **ADC_mean_** | AUC  median (2.5^th^ percentile – 97.5^th^ percentile) | 0.953 (0.922 - 0.975) | 0.931  (0.874 - 0.967) | 0.949  (0.914 - 0.972) |
|  | Cutoff maximizing Youden’s index  median (2.5^th^ percentile – 97.5^th^ percentile) | 1.058 x 10^-3^mm²/s  (0.959 – 1.166) | 1.052 x 10^-3^mm²/s  (0.928 – 1.198) | 1.070 x 10^-3^mm²/s (0.961 – 1.188) |
| **Short axis diameter** | AUC  median (2.5^th^ percentile – 97.5^th^ percentile) | 0.671  (0.601 - 0.736) | 0.640  (0.539 - 0.733) | 0.548  (0.474 - 0.623) |
|  | Cutoff maximizing Youden’s index  median (2.5^th^ percentile – 97.5^th^ percentile) | 7.309 mm (5.786 - 9.190) | 7.280 mm  (5.202 - 10.190) | 6.520 mm (4.550 - 9.221) |

**Supplemental References**

1 Brown H, Prescott R (2015) Applied Mixed Models in Medicine, 3 edn. John Wiley & Sons Ltd, New York

2 Carrasco JL, Jover L (2003) Estimating the generalized concordance correlation coefficient through variance components. Biometrics 59:849-858

3 Nakagawa S, Schielzeth H (2010) Repeatability for Gaussian and non-Gaussian data: a practical guide for biologists. Biological reviews of the Cambridge Philosophical Society 85:935-956

4 Faraway JJ (2016) Extending the Linear Model with R - Generalized Linear, Mixed Effects and Nonparametric Regression Models, 2 edn. CRC Press LLC, Boca Raton, Florida

5 Altman DG, Bland JM (1983) Measurement in medicine: The analysis of method comparison studies. Journal of the Royal Statistical Society, Series D (The Statistician) 32:307-317

6 Parker RA, Weir CJ, Rubio N et al (2016) Application of Mixed Effects Limits of Agreement in the Presence of Multiple Sources of Variability: Exemplar from the Comparison of Several Devices to Measure Respiratory Rate in COPD Patients. PLoS One 11:e0168321

7 R Core Team (2022) R: A Language and Environment for Statistical Computing. R Foundation for Statistical Computing, Vienna, Austria. Available via <https://www.R-project.org/>

8 Bates D, Mächler M, Bolker B, Walker S (2015) Fitting Linear Mixed-Effects Models Using lme4. Journal of Statistical Software 67:1-48
